# Cardiovascular magnetic resonance feature tracking for characterization of patients with heart failure with preserved ejection fraction: correlation of global longitudinal strain with invasive diastolic functional indices

**DOI:** 10.1186/s12968-020-00636-w

**Published:** 2020-06-04

**Authors:** Haruno Ito, Masaki Ishida, Wakana Makino, Yoshitaka Goto, Yasutaka Ichikawa, Kakuya Kitagawa, Taku Omori, Kaoru Dohi, Masaaki Ito, Hajime Sakuma

**Affiliations:** 1 Departments of Radiology, Mie University Hospital, 2-174 Edobashi, Tsu, Mie 514-8507 Japan; 2 Departments of Cardiology and Nephrology, Mie University Hospital, 2-174 Edobashi, Tsu, Mie 514-8507 Japan

**Keywords:** Cardiovascular magnetic resonance, Heart failure with preserved ejection fraction, Feature tracking, Global longitudinal strain, Extracellular volume fraction

## Abstract

**Background:**

Left ventricular (LV) diastolic dysfunction is the main cause of heart failure with preserved ejection fraction (HFpEF), and is characterized by LV stiffness and relaxation. Abnormal LV global longitudinal strain (GLS) is frequently observed l in HFpEF, and was shown to be useful in identifying HFpEF patients at high risk for a cardiovascular event. Cardiovascular magnetic resonance (CMR) feature tracking (CMR-FT) enables the reproducible and non-invasive assessment of global strain from cine CMR images. However, the association between GLS and invasively measured parameters of diastolic function has not been investigated. We sought to determine the prevalence and severity of GLS impairment in patients with HFpEF by using CMR-FT, and to evaluate the correlation between GLS measured by CMR-FT and that measured by invasive diastolic functional indices.

**Methods:**

Eighteen patients with HFpEF and 18 age- and sex-matched healthy control subjects were studied. All subjects underwent cine, pre- and post-contrast T1 mapping and late gadolinium-enhancement CMR. In the HFpEF patients, invasive pressure–volume loops were obtained to evaluate LV diastolic properties. GLS was quantified from cine CMR, and extracellular volume fraction (ECV) was quantified from pre- and post-contrast T1 mapping as a known imaging biomarker for predicting LV stiffness.

**Results:**

GLS was significantly impaired in patients with HFpEF (− 14.8 ± 3.3 vs.–19.5 ± 2.8%, *p* < 0.001). Thirty nine percent (7/18) of HFpEF patients showed impaired GLS with a cut-off of − 13.9%. Statistically significant difference was found in ECV between HFpEF patients and controls (32.2 ± 3.8% vs. 29.9 ± 2.6%, *p* = 0.044). In HFpEF patients, the time constant of active LV relaxation (Tau) was strongly correlated with GLS (r = 0.817, *p* < 0.001), global circumferential strain (GCS) (r = 0.539, *p* = 0.021) and global radial strain (GRS) (r = − 0.552, *p* = 0.017). Multiple linear regression analysis revealed GLS as the only independent predictor of altered Tau (beta = 0.817, *p* < 0.001) among age, LV end-diastolic volume index, LV end-systolic volume index, LV mass index, GCS, GRS and GLS.

**Conclusions:**

CMR-FT is a noninvasive approach that enables identification of the subgroup of HFpEF patients with impaired GLS. CMR LV GLS independently predicts abnormal invasive LV relaxation index Tau measurements in HFpEF patients. These findings suggest that feature-tracking CMR analysis in conjunction with ECV, may enable evaluation of diastolic dysfunction in patients with HFpEF.

## Background

Heart failure with preserved ejection fraction (HFpEF) is a prevalent and growing public health problem [1]. Although the pathophysiology of HFpEF is multifactorial, left ventricular (LV) diastolic dysfunction, which is characterized by LV stiffness and relaxation, is recognized as the main cause [2–4]. A previous study demonstrated that LV extracellular volume fraction (ECV) is a noninvasive indicator of LV stiffness in patients with HFpEF [5]. A more recent study employing speckle-tracking echocardiography found that systolic function measures such as LV global longitudinal strain (GLS) are frequently abnormal in HFpEF patients [6]. A recent study by Shah et al. also indicated that abnormal GLS is of value to identify patients with HFpEF at high risk for a cardiovascular event [7]. The cardiovascular magnetic resonance (CMR) feature tracking (FT; CMR-FT) technique enables the reproducible assessment of GLS from routine clinical CMR images with reduced observer dependency as compared to echocardiography [8]. However, the association between GLS determined by CMR-FT and the indices of diastolic function determined by cardiac catheterization has not been fully investigated in HFpEF patients.

Consequently, the purposes of this study were to determine the prevalence and severity of GLS impairment in patients with HFpEF by using CMR-FT and to evaluate the relationship between CMR-FT GLS and diastolic functional indices determined by invasive catheterization.

## Methods

### Patient population

**Fig. 1:**
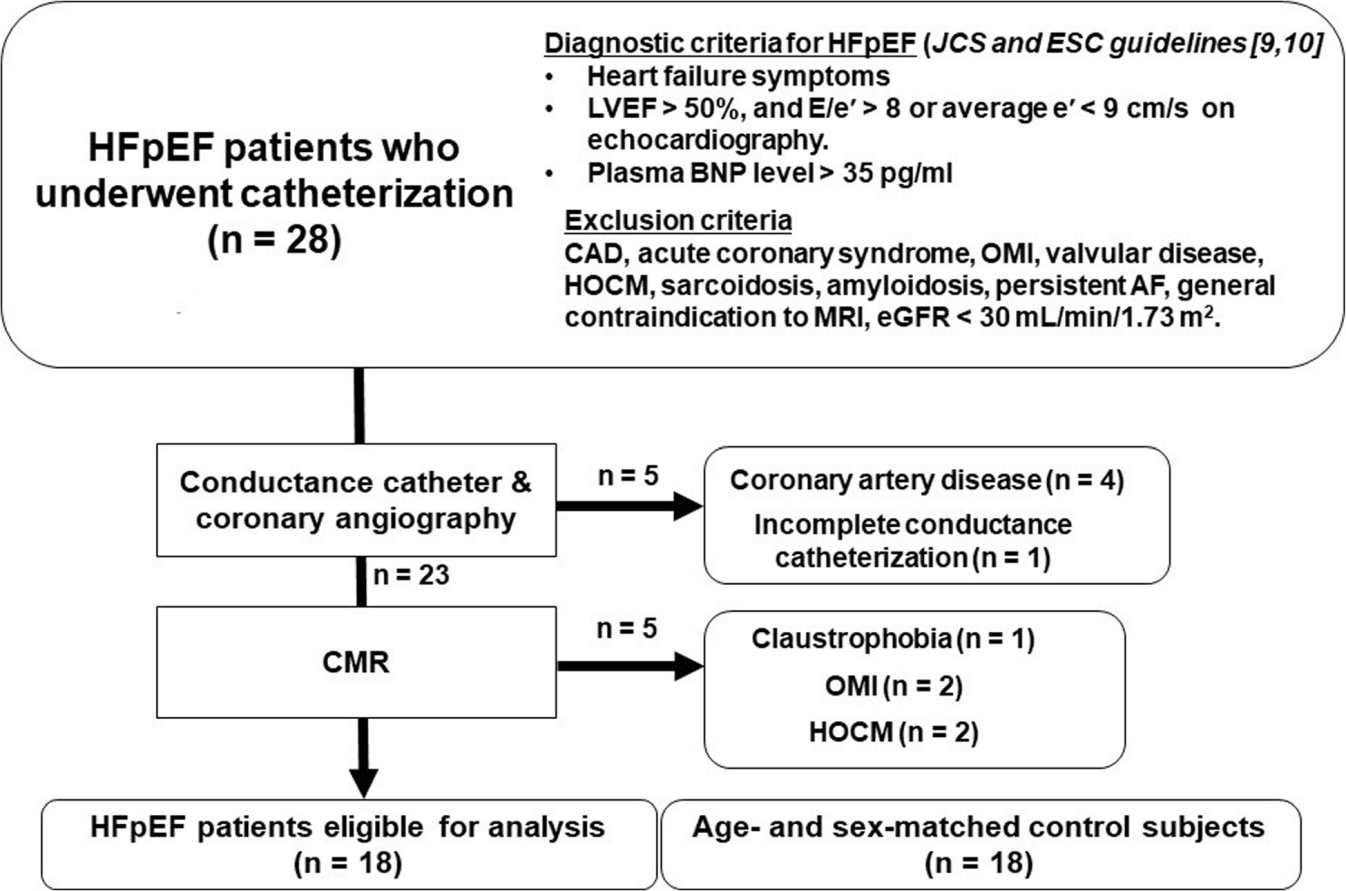
Subject selection. Of 28 patients with HFpEF who underwent CMR and invasive catheterization, 5 were excluded due to significant CAD on CAG, 1 due to claustrophobia, 2 due to OMI on CMR, and 2 due to HOCM on CMR

Twenty-eight patients with HFpEF who underwent invasive cardiac catheterization were enrolled. HFpEF was diagnosed in accordance with the following criteria: LV ejection fraction (EF) ≥50% as measured by echocardiography; New York Heart Association functional class ≥II; either E/e′ > 8 or average e′ < 9 cm/s on echocardiography, and plasma brain natriuretic peptide (BNP) level > 35 pg/mL [9, 10]. Exclusion criteria were coronary artery disease, acute coronary syndrome, prior myocardial infarction, greater than moderate valvular disease, hypertrophic obstructive cardiomyopathy, sarcoidosis, amyloidosis, persistent atrial fibrillation, general contraindication to CMR and an estimated glomerular filtration rate (eGFR) < 30 mL/min/1.73 m^2^. The exclusion criteria are listed in Fig. 1. Consequently, 18 patients (65 ± 17 years; 3 males) with HFpEF who completed invasive catheterization and CMR were eligible. This population was compared with 18 age- and sex-matched healthy control subjects (61 ± 14 years; 7 males).

This study was conducted in accordance with the principles of the Declaration of Helsinki and with the approval of our Institutional Review Board (reference number 2742). All participants gave written, informed consent prior to participation in this study.

### CMR imaging

CMR studies were performed on a 3 T CMR scanner (Ingenia, Philips Healthcare, Best, The Netherlands) using dS coils for signal reception. The CMR study protocol included cine CMR, native T1 mapping using a modified Look-Locker inversion recovery (MOLLI) sequence, late gadolinium enhancement (LGE) CMR and post-contrast T1 mapping using MOLLI. Cine CMR images were acquired with retrospective electrocardiographic gating and a segmented balanced steady-state free precession sequence during brief periods of breath-holding at a shallow expiration in the following planes: LV 2-chamber and 4-chamber views and short-axis planes covering the entire left ventricle and right ventricle (repetition time (TR), 3.2 ms; echo time (TE), 1.6 ms; flip angle (FA), 55°; field of view (FOV), 350 × 350 mm^2^; acquisition matrix, 176 × 306; reconstruction matrix, 352 × 352; slice thickness, 10 mm; sensitivity encoding (SENSE) factor, 3; temporal resolution, 58 ± 11 ms; number of phases per cardiac cycle, 20) [11]. All cine images were acquired with 20 phases per cardiac cycle. The short-axis plane was defined as the plane perpendicular to the horizontal and vertical long-axis views. T1 mapping was performed using a 17-heartbeat steady-state free procession 3–3-5 MOLLI sequence on the short-axis imaging plane at the level of the LV base, midLV and apical levels (TR, 2.6 ms; TE, 1.1 ms; FA, 35°; FOV, 300 × 330 mm^2^; acquisition matrix, 176 × 141; reconstruction matrix, 288 × 288; slice thickness, 10 mm; SENSE factor, 2) [12]. At 5–10 min after bolus administration of gadoterate meglumine (Gd-DOTA, Magnescope®; Guerbet Japan, Tokyo, Japan) (cumulative dose of 0.15 mmL/kg), short- and long-axis 2D inversion recovery LGE images were acquired with an inversion recovery gradient-echo imaging sequence. Post-contrast MOLLI T1 mapping was repeated as for native T1 mapping [12].

### CMR image analysis

CMR image analyses were carried out using CMR analysis software, cvi42 (Circle Cardiovascular Imaging Inc., Calgary, Canada) by an experienced radiologist (HI, 3 years of CMR experience) who was blinded to the subjects’ clinical information and the results of other diagnostic tests.

LV volume and function were analyzed based on the short-axis cine stack. The endocardial and epicardial borders of the LV wall were manually traced on cine CMR images in the end-diastolic and end-systolic phases. LV mass was calculated as the volume of the LV myocardium multiplied by the specific gravity of the myocardium (1.05 g/mL). Right ventricular (RV) volume and function were then analyzed based on the short-axis cine stack. LV and RV measurements were indexed to body surface area (BSA). To assess the time course of global volumetric filling, LV endocardial and epicardial contouring was added for all LV short-axis slices across all temporal phases. Peak filling rate (PFR) was defined as the maximal change in LV volume between sequential temporal phases (Δ volume/Δ phase) [13, 14]. This index was normalized for LV end-diastolic volume (nPFR) [15]. Left atrial (LA) volume was measured using the biplane area–length method, employing 2- and 4-chamber views [16]. Right atrial (RA) volume was measured using the single-plane area–length method in the 4-chamber view [17]. The maximal and minimal atrial volume was measured to calculate atrial ejection fraction.

LV and RV strain analysis was performed by a feature-tracking algorithm [18]. The endocardial and epicardial borders of myocardium were manually traced in the end-diastolic phase of 2- and 4-chamber view cine CMR images for LV GLS and a 4-chamber view cine CMR image for RV GLS. The software then automatically propagated the endocardial and epicardial contours and tracked the motion of the in-plane tissue voxels through the entire cardiac cycle. In addition, global circumferential strain (GCS) and global radial strain (GRS) was determined using short-axis cine CMR covering entire LV. Consequently, peak GLS, GCS and GRS were recorded for LV and peak GLS for RV [19]. LA myocardial feature tracking was performed, in which the LA endocardial and epicardial borders were manually traced in the 4-chamber view and an automated tracking algorithm was applied [20]. Tracking was repeated three times and the averages of these repetitions were used for further analyses [20].

T1 measurement was performed by pixel-wise quantification [12]. Respiratory motion in the images was corrected for by non-rigid image registration before T1 maps were generated by fitting pixels to the equation s(t) = a–b exp.(−t/T1*), and T1 = T1*(b/a–1), where a and b are constants, t is time, and s(t) is signal intensity at time t. The generated native and post-contrast T1 maps were stored in Digital Imaging and Communications in Medicine (DICOM) format. Native T1 values were averaged for the T1 value per pixel of the LV blood pool and the myocardium, determined on regions of interest manually drawn in the center of the blood pool and the LV myocardium, for each of the three LV short-axis images, before and after contrast administration. A pixel-wise extracellular volume fraction (ECV) map was then generated based on the combined pre- and post-contrast T1 maps using the formula: ECV = (∆R1myocardium/∆R1blood) × (1–hematocrit), where R1 = 1/T1. ECV values were averaged for all pixels.

### Cardiac catheterization protocol

Invasive cardiac catheterization was performed with right femoral artery access. To measure both LV volume and pressure simultaneously, a 6F single-field conductance catheter (Webster Laboratories, Baldwin Park, California, USA) with a 2F microtip manometer (Millar Instruments, Inc., Houston, Texas,USA) or a coronary-pressure guidewire (Philips Volcano, CA, USA) placed within its lumen was advanced to the LV apex and connected to a digital stimulator microprocessor (Sigma V [dual-field system]; Leycom, Zoetermeer, The Netherlands). The conductance catheter technique and its principles have been fully described previously [21–23]. Calibration offset (parallel conductance) was corrected by matching the conductance catheter signal at end-diastole with the end-diastolic volume measured by cine CMR.

An experienced cardiologist without knowledge of other test results analyzed the conductance catheter data and invasive angiography data. The monoexponential-based time constant (Tau) of isovolumetric fall of LV pressure was calculated assuming that pressure decayed to a non-zero asymptote [24]. The diastolic pressure–volume relation is described by the exponential eq. P = P0 + α(ℯβV − 1), where P is LV pressure, P0 is pressure offset, α is a curve-fitting constant, V is LV volume and β is a load-independent constant used to quantify passive stiffness of the LV chamber [25].

### Statistical analysis

Statistical analyses were performed using SPSS (v 19.0; Statistical Package for the Social Sciences, International Business Machines, Inc., Armonk, New York, USA). Normality of continuous variables was assessed using the Shapiro–Wilk test. As all continuous variables were normally distributed, data for continuous variables are presented as the mean ± standard deviation (SD). Categorical variables are presented as frequencies and percentages. Comparisons between groups were made using unpaired Student’s *t* test for continuous variables and chi-square tests or Fisher exact test for categorical variables. Univariate and stepwise multivariate linear regression analyses were performed to identify predictors of Tau. Any variable with a *p* value < 0.10 in a univariate analysis was included in a subsequent multivariable model. Pearson’s correlation coefficient was used to measure linear correlations between two variables. Statistical significance was defined as *p* < 0.05.

## Results

The baseline characteristics of the 18 HFpEF patients and the 18 healthy control subjects are summarized in Table 1. HFpEF patients were more likely to have hypertension and diabetes compared with the control subjects. A more frequent use of antihypertensive and heart failure medications was noted in HFpEF patients compared with the controls.

**Table 1:** Baseline characteristics

	HFpEF (*n* = 18)	Healthy Controls (*n* = 18)	p
Age (years)	65 ± 17	61 ± 14	0.414
Male	3 (16.7)	7 (38.9)	0.137
BMI (kg/m^2^)	25.8 ± 6.7	22.7 ± 3.9	0.098
BNP (pg/mL)	178 ± 319	n/a	n/a
NYHA I	0 (0)	18 (100)	< 0.0001
NYHA II	11 (61.1)	0 (0)	0.0002
NYHA III	6 (33.3)	0 (0)	0.013
NYHA IV	1 (5.6)	0 (0)	0.473
Smoker	5 (27.8)	4 (22.2)	0.700
Hypertension	15 (83.3)	0 (0)	< 0.001
Dyslipidemia	8 (44.4)	5 (27.8)	0.298
Diabetes	2 (11.1)	0 (0)	0.488
Beta-blocker	8 (44.4)	1 (5.6)	0.070
ACE/ARB	12 (66.7)	0 (0)	< 0.001
Calcium channel blocker	8 (44.4)	0 (0)	0.003
Diuretics	8 (44.4)	0 (0)	0.003
Statins	3 (16.7)	3 (16.7)	1.000

*BMI* Body mass index, *BNP* Brain natriuretic peptide, *NYHA* New York Heart Association, *ACE* Angiotensin converting enzyme inhibitor, *ARB* Angiotensin receptor blocker

### CMR results

**Fig. 2:**
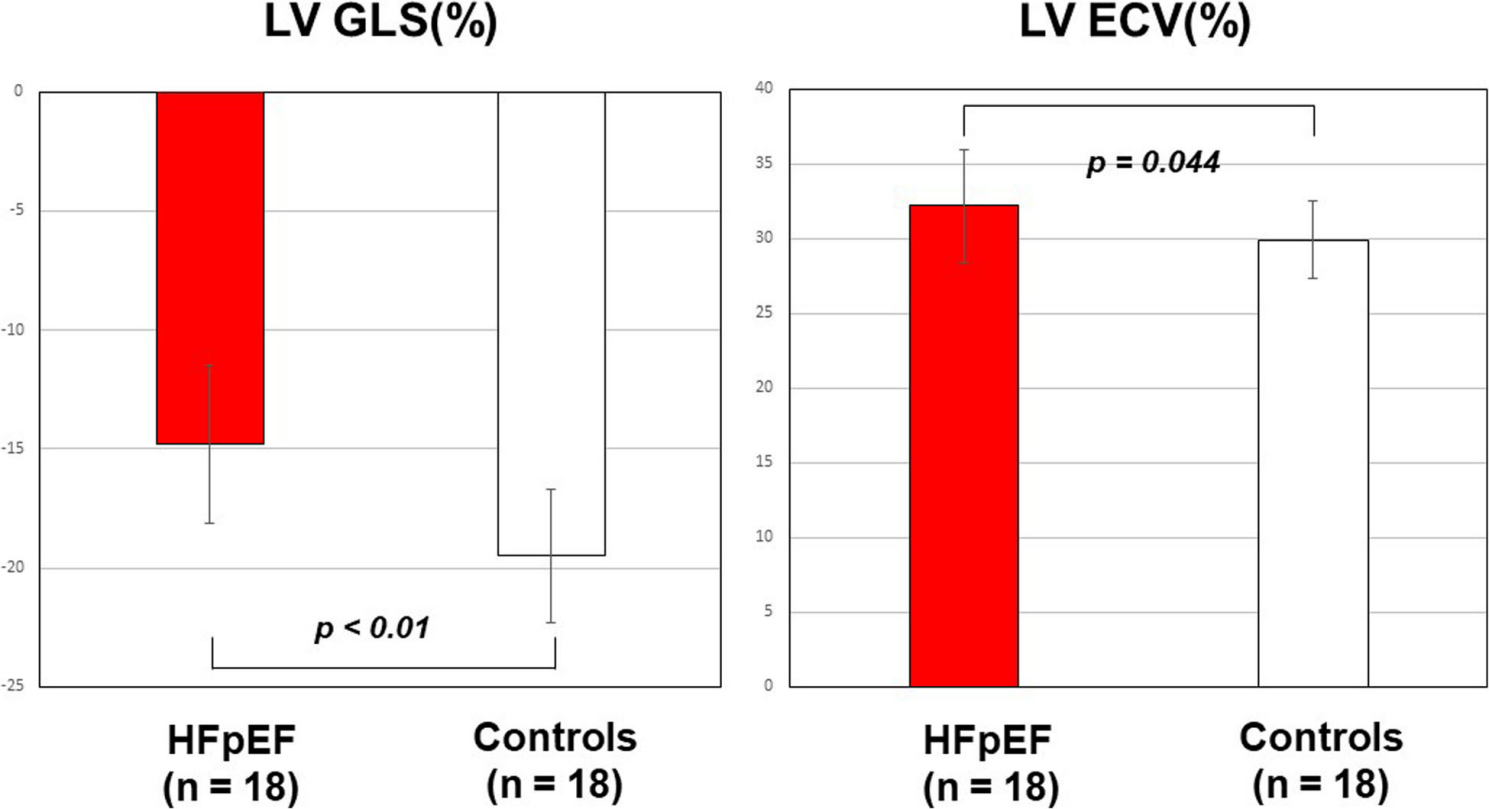
Differences in LV GLS and LV ECV between HFpEF patients and healthy controls. LV GLS was significantly impaired in HFpEF patients compared with controls (− 15.2 ± 4.0%, − 19.1 ± 1.8%, *p* = 0.0005) whereas LV ECV was not significantly different between HFpEF patients and controls (32.2 ± 3.7% vs. 29.7 ± 3.9%, *p* = 0.057)

**Fig. 3:**
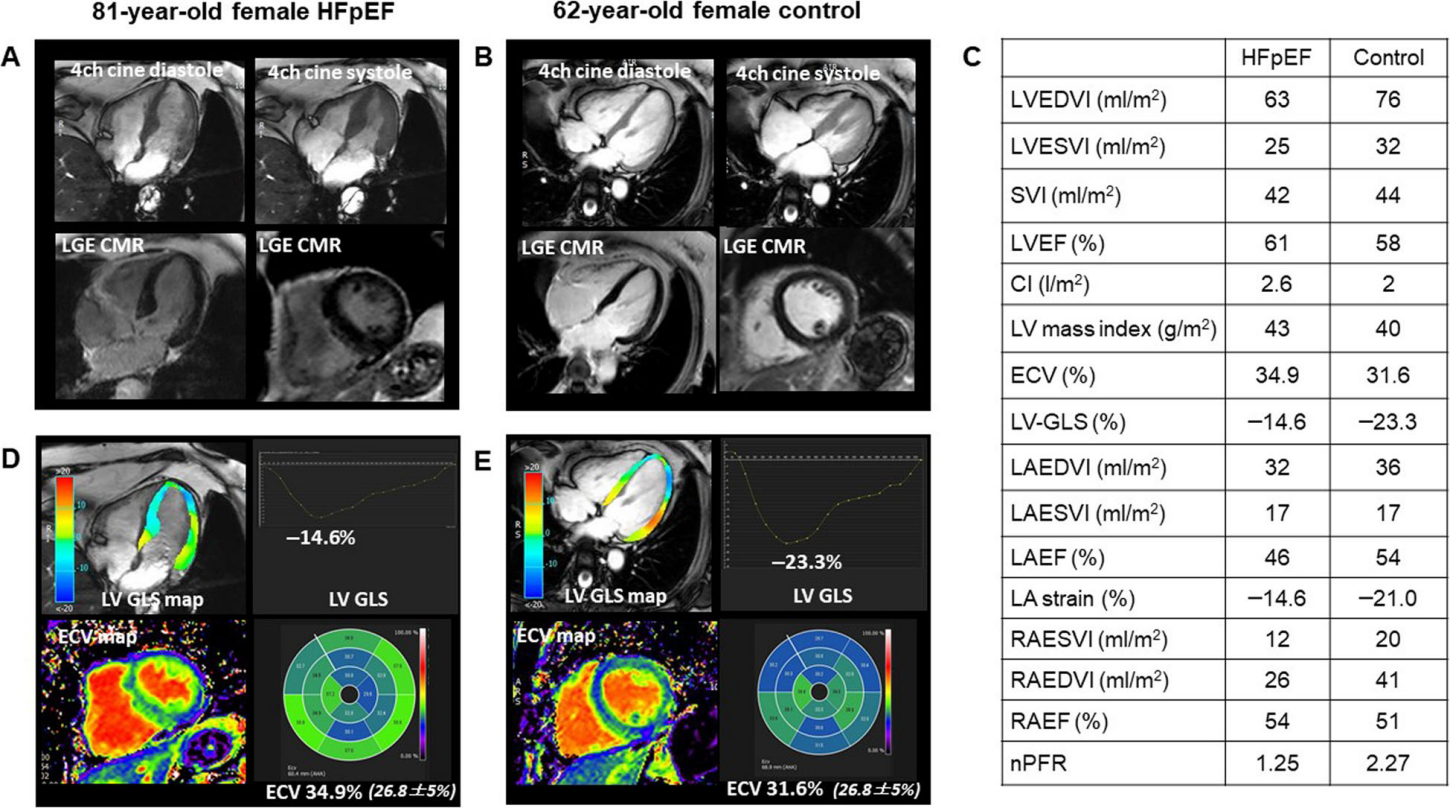
Representative imaging findings in a patient with HFpEF and a healthy control subject. Visual assessment of cine and LGE CMR images shows no abnormal findings in images of an 81-year-old female with HFpEF (**a**) or a 62-year-old healthy female control (**b**). LV volume, EF and mass are normal in both subjects (**c**). However, LV GLS is substantially lower (− 14.6%) and ECV is higher (34.9%) in the patient with HFpEF (**d**) compared with the control subject (− 23.3 and 31.6%, respectively) (**e**)

**Fig. 4:**
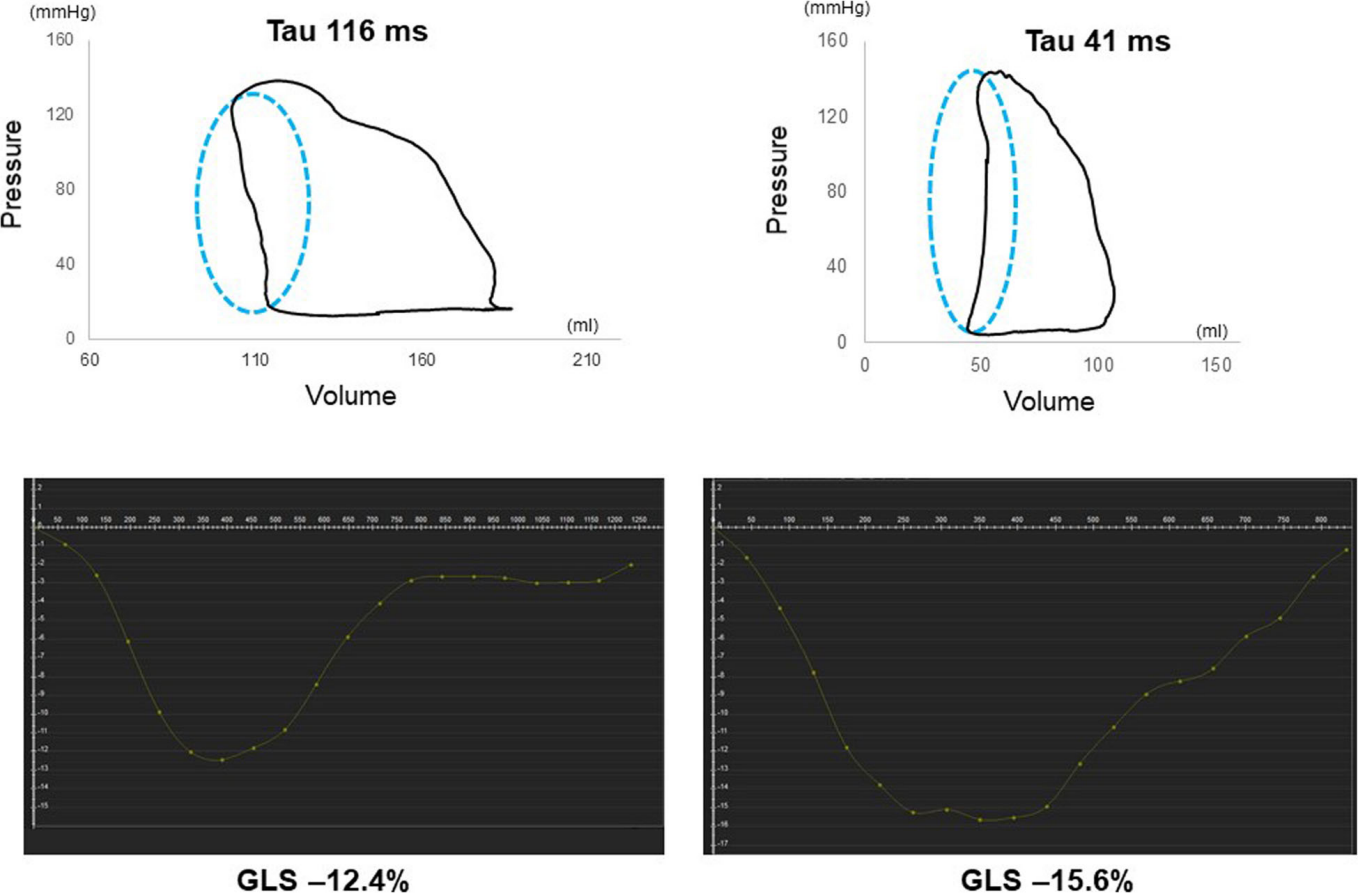
The examples of pressure volume loop in HFpEF patients with long Tau (left) and short Tau (right) with corresponding GLS curves. The patients with longer Tau had more impaired GLS

All 18 patients with HFpEF and the 18 healthy control subjects had complete CMR data. HFpEF patients had more impaired LV GLS (− 14.8 ± 3.3% vs. –19.5 ± 2.8%, *p* < 0.001) (Fig. 2), GCS (*p* = 0.004) and GRS (*p* = 0.007). Thirty nine percent (7/18) of HFpEF patients showed impaired GLS with a cut-off of − 13.9% (mean + 2SD of controls). In addition as demonstrated in Table 2, HFpEF patients had a larger LV mass index (*p* = 0.02) and LV end-systolic volume index (ESVI; *p* = 0.036), more impaired LA total strain (*p* = 0.042), smaller LA EF (*p* < 0.001) and RA EF (*p* = 0.001), larger LA end-diastolic volume index (EDVI) (*p* = 0.007), LA ESVI; *p* < 0.001) and RA ESVI (*p* = 0.003), more impaired nPFR (*p* < 0.001) and higher ECV (*p* = 0.044). Representative cases are shown in Figs. 3 and 4.

**Table 2:** Cardiovascular magnetic resonance data

	HFpEF (n = 18)	Healthy Control (n = 18)	p
LV EDVI (mL/m^2^)	87.9 ± 21.3	79.1 ± 18.1	0.189
LV ESVI (mL/m^2^)	37.9 ± 13.9	29.6 ± 7.8	0.036
LV EF (%)	57.6 ± 7.7	59.4 ± 14.4	0.646
LV CI (L/min/m^2^)	3.27 ± 1.16	5.0 ± 8.21	0.381
LV mass index (g/m^2^)	63.1 ± 30.7	43.9 ± 10.4	0.020
LV ECV (%)	32.2 ± 3.8	29.9 ± 2.6	0.044
LV GLS (%)	−14.8 ± 3.3	−19.5 ± 2.8	< 0.001
LV GCS (%)	− 18.7 ± 4.2	−22.5 ± 3.0	0.004
LV GRS (%)	39.0 ± 14.6	50.9 ± 9.2	0.007
nPFR	1.82 ± 0.50	2.76 ± 0.77	< 0.001
LA EDVI (mL/m^2^)	45.8 ± 15.7	33.1 ± 10.1	0.007
LA ESVI (mL/m^2^)	25.7 ± 10.7	13.1 ± 5.4	< 0.001
LA EF (%)	44.7 ± 6.5	60.9 ± 6.2	< 0.001
LA total strain (%)	15.7 ± 5.1	18.9 ± 3.6	0.042
LA conduit strain (%)	8.7 ± 4.0	10.3 ± 3.0	0.178
RV EDVI (mL/m^2^)	66.6 ± 14.8	78.2 ± 19.6	0.052
RV ESVI (mL/m^2^)	23.1 ± 9.1	30.1 ± 11.7	0.057
RV EF (%)	65.5 ± 9.3	62.2 ± 9.4	0.301
RV GLS (%)	−20.9 ± 4.4	−21.6 ± 4.4	0.643
RA EDVI (mL/m^2^)	34.0 ± 12.7	28.5 ± 9.2	0.142
RA ESVI (mL/m^2^)	21.0 ± 9.3	13.1 ± 4.5	0.003
RA EF (%)	37.8 ± 13.6	52.9 ± 12.3	0.001

*LV* Left ventricular, *EDVI* End-diastolic volume index, *ESVI* End-systolic volume index, *EF* Ejection fraction, *CI* Cardiac index, *ECV* Extracellular volume fraction, *GLS* Global longitudinal strainm, *GCS* Global circumferential strain, *GRS* Global radial strain, *LA* Left atrial, *RA* Right atrial, *nPFR* Normalized peak filling rate

### Correlation of measures of diastolic function

The conductance catheter results are listed in Table 3. Tau and β were 71.4 ± 27.9 ms and 0.051 ± 0.011, respectively.

**Table 3:** Conductance catheter data

	HFpEF (n = 18)
HR, beats/min	66.6 ± 7.8
LV ESP, mmHg	148.9 ± 34.4
LV EDP, mmHg	15.2 ± 8.3
Tau, ms	71.4 ± 27.9
LV ESPVR, mmHg/mL	2.0 ± 1.3
Ea, mmHg/mL	2.1 ± 1.0
β	0.051 ± 0.011

*HR* Heart rate, *ESP* End-systolic pressure, *EDP* End-diastolic pressure, *ESPVR* End-systolic pressure–volume relationship, *Ea* Arterial elastance

**Fig. 5:**
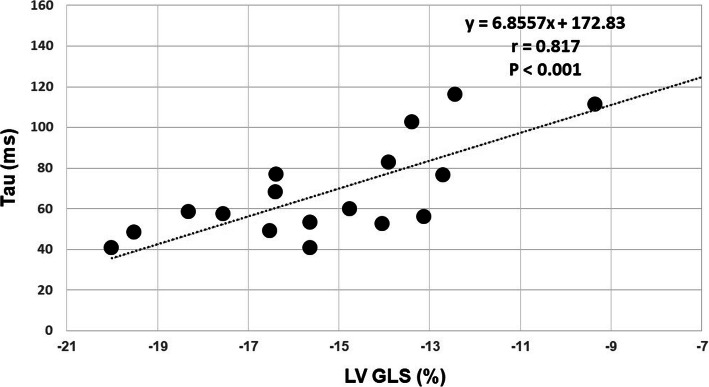
Correlation of LV relaxation and GLS. A significant positive correlation was observed between LV relaxation (Tau) and LV GLS (r = 0.817, *p* < 0.001)

Univariate linear regression analyses showed that, among age, CMR parameters and β, Tau was significantly correlated with age (r = − 0.676, *p* = 0.002), LV EDVI (r = 0.536, *p* = 0.022), LV ESVI (r = 0.486, *p* = 0.041), LV mass index (r = 0.504, *p* = 0.033), LV GLS (r = 0.817, *p* < 0.001) (Fig. 5), LV GCS (r = 0.539, *p* = 0.021) and LV GRS (r = − 0.552, *p* = 0.017) (Table 4). On step-wise multivariate linear regression analyses that included age, LV EDVI, LV ESVI, LV mass index, LV GRS, LV GCS and LV GLS as variables, LV GLS was the only independent predictor of Tau (beta = 0.817, *p* < 0.001).

**Table 4:** Correlation of various factors with the time constant of active relaxation (Tau)

	Pearson’s correlation coefficient (r)	B _non-standardized_	p
Age	−0.676	−1.118	0.002
BMI	0.102	0.424	0.687
LV EDVI	0.536	0.703	0.022
LV ESVI	0.486	0.978	0.041
LV CI	0.067	1.622	0.791
LV EF	−0.244	−88.398	0.328
LV mass index	0.504	0.458	0.033
LV ECV	−0.064	− 0.467	0.801
LV GLS	0.817	6.141	< 0.001
LV GCS	0.539	−3.795	0.021
LV GRS	−0.552	−1.411	0.017
nPFR	−0.015	−0.845	0.953
LA EDVI	0.219	0.390	0.383
LA ESVI	0.141	0.367	0.577
LA EF	0.068	29.204	0.788
LA total strain	−0.061	−0.339	0.816
LA conduit strain	0.195	1.382	0.452
RV EDVI	0.36	0.679	0.142
RV ESVI	0.104	0.320	0.683
RV EF	0.234	70.330	0.351
RV GLS	0.412	2.639	0.089
RA EDVI	−0.169	−0.372	0.504
RA ESVI	−0.124	− 0.371	0.625
RA EF	−0.106	−21.681	0.676
β	0.106	226.886	0.686

Univariate regression analysis was performed with the Pearson correlation coefficient

## Discussion

The main finding of this study is that among HFpEF patients CMR-FT GLS is independently associated with invasive measures of LV relaxation (Tau). Diastolic dysfunction is the hemodynamic consequence of the pathologies involved in HFpEF [5]. Prolongation of active myocardial relaxation and an increase in load-independent LV stiffness have been reported as the main mechanisms for diastolic dysfunction [5]. Therefore, the present finding suggests that systolic longitudinal dysfunction is closely associated with diastolic dysfunction in HFpEF patients. Previous studies have reported an inverse correlation between LV relaxation and LV contractility [26, 27]. According to those studies, the mechanism of the close association between systolic LV longitudinal dysfunction and impaired LV relaxation in HFpEF patients can be attributed to the elastic recoil of the LV myocardium [26]. During systole the myocardial wall stores energy in the form of elastic recoil, and this energy is released when the myocardium relaxes [26]. Thus, strain measurements by CMR-FT may enable the detection of diastolic dysfunction in the absence of an overt reduction in LV EF in HFpEF patients. It is recognized that endocardial dysfunction leads to depressed GLS and the preserved GCS and LV torsion usually compensates for the depressed GLS in HFpEF [28]. In our study, Tau had stronger association with GLS than GCS or GRS, suggesting that LV relaxation are closely associated with endocardial dysfunction. Furthermore, GLS measurement by CMR-FT is reproducible, easy to perform and less time consuming. GLS can be a meaningful tool in the routine clinical practice for patients with HFpEF.

LV stiffness is thought to be a consequence of an increase in extracellular matrix, reflecting abnormal diffuse myocardial fibrosis. In a recent study by Rommel et al., multivariate analysis revealed ECV as the only independent predictor of the myocardial stiffness constant (β), suggesting that in HFpEF patients with elevated ECV, the dominant pathomechanism is an increase in LV stiffness [5]. Rommel et al. mentioned that in addition to myocardial stiffness, impairment of active relaxation may be the important pathomechanism in HFpEF patients too. The findings of our study support their hypothesis. In our study, ECV value in controls was relatively high as compared to the previous studies. This might be because the mean age of controls was relatively high (61 ± 14 years) and 61% were female in which the ECV tends to demonstrate higher value.

In the present study, 39% of HFpEF patients showed impaired GLS with a value of less than − 13.9% (mean + 2SD of controls in our study). A recent study by Shah et al. using echocardiography reported impaired GLS as an independent imaging biomarker for identifying patients with HFpEF at high risk for cardiovascular morbidity and mortality [7], with a cut-off of − 15.8%. CMR-FT may also be able to identify HFpEF patients at high risk for a cardiovascular event; however, further study is required to establish the ideal cut-off value for CMR-FT. Another recent CMR study has shown that high ECV is associated with higher rates of morbidity and mortality in patients with HFpEF [29]. Furthermore, the study conducted by Mordi et al. demonstrated that echo-derived GLS and CMR-derived ECV are able to independently discriminate between hypertensive heart disease and HFpEF and identify patients with prognostically significant functional limitations [30]. In a similar manner, it is considered that measurement of LV strain and ECV by the CMR-only approach may provide two independent parameters of LV relaxation and stiffness that reflect the degree of diastolic dysfunction and may also have the prognostic implications in HFpEF patients. Further studies are needed to confirm the value of the measurement of LV strain and ECV by the CMR-only approach.

LA dysfunction is common in HFpEF because it is linked with LV dysfunction [20, 31]. Significant impairment of LA total strain in HFpEF patients was found in the present study, and LA total strain has recently been identified as a powerful prognostic factor in HFpEF patients [32]. Schuster et al. has shown that in the survivors of acute myocardial infarction LA total strain has incremental prognostic value in addition to any CMR measurements [33]. LA total strain might have incremental value for stratifying HFpEF patient prognosis, in addition to LV GLS and ECV. However, LA strain did not show the significant correlation with invasive measures of diastolic dysfunction in the current study and previous study [20], suggesting that LA strain parameters might offer exclusive clinical information which is only possible non-invasively. LA conduit function is closely related to LV stiffness [34]. von Roder et al. demonstrated that LA conduit strain was significantly impaired in HFpEF patients than controls and was the strongest predictor of exercise capacity [20]. However, in the present study, significant difference was not observed in LA conduit strain between HFpEF patients and control subjects. It might be speculated that LV stiffness in our patients was milder than the patients in the study by von Roder et al. Interestingly, Kowallick et al. demonstrated that LA conduit functions evaluated by CMR-FT can make discrimination among a hypertrophied phenotype, HFpEF and volunteers, as demonstrated by Mordi et al. where the discrimination between HFpEF and hypertensive heart disease was achieved based on GLS and ECV [35]. In the present study, there was no difference in RV volume or function between HFpEF patients and controls, whereas RA EF was significantly impaired in HFpEF patients compared with the controls. Recent study demonstrated that RV systolic function was preserved while RV early filling was impaired and compensated by increased RA booster pump function in compensated HFpEF patients [36]. Although it is difficult to interpret clinical implications of our finding at the present time, RV systolic function can be preserved with the impairment of RA function parameters in a certain condition. Future study focusing on RV and RA function in HFpEF patients is warranted.

The values of Tau and β in HFpEF patients were substantially different between our study and Rommel’s study. The reason for the difference may be due to the difference in the fitting equations to determine those values. In our study the best fit method assuming that pressure decayed to a non-zero asymptote was used for calculating Tau (*P = P*_*0*_*e*^*-t/Tau*^ *+ P*_*B*_) [24], while Rommel et al. employed Weiss’s method (P = e^At + B^) (asymptote = 0) [37]. To determine β, the formula of “*P* = *A e*^β*V*^” was used in Rommel’s study [38], whereas the formula of “*P* = *A (e*^β*V*^*-1)*” was used in the present study [25], where *P* is the LV pressure, *V* is the LV volume, *A* is a curve fitting constant. Consequently, our method provide larger Tau and smaller β compared to Rommel’s method. LV pressure volume analysis has remained a more research-based reference standard for confirming definite evidence of HFpEF due to its invasive nature. The pulmonary wedge pressure (PCWP) during physiological exercise emerged as the clinical reference standard to define HFpEF [39], which is clinically beneficial as it can avoid the risk of conventional LV pressure volume analysis. However, the study investigating the association between exercise PCWP and CMR functional parameters is still lacking. Further study will be needed.

In our study, cine images consisted of 20 phases per cardiac cycle. Cine images with lower temporal resolution are more prone to miss the short-lived events during the isovolumic period. However, longer breath-hold duration is required to obtain higher temporal resolution in cine CMR imaging. In this respect, typically cine CMR with 20–30 phases per cardiac cycle was used in the most of previous studies [15, 18, 20, 40, 41]. CMR-FT using cine images consisting of 20–30 phases per cardiac cycle substantially underestimated true GLS [42].However, the previous studies and our study successfully demonstrated that cine images with 20–30 phases per cardiac cycle can provide useful information in a clinical setting [15, 18, 20, 40, 41].

Echocardiography enables the noninvasive identification of diastolic dysfunction based on transmitral inflow (E and A values) or on myocardial compliance sampled at regional myocardial locations (e′) [43]. The global LV filling curves of cine CMR provide an alternative means of assessing diastolic physiology based on the timing and pattern of dynamic changes in LV chamber volume [13, 14]. The LV filling curve is usually transformed to the first derivative to obtain the early filling profile (i.e., PFR), which corresponds to the E value measured by echocardiography. Because it is influenced by filling pressure as well as inversely altered by changes in relaxation [26], E is usually corrected for the influence of relaxation (e′) in echocardiography (E/e′). In agreement with previous studies, in the present study PFR normalized by LV end-diastolic volume (nPFR) was significantly impaired in HFpEF patients compared with controls [13, 14]. However, the present study found no association of PFR or nPFR with Tau, which can be attributed to the load dependence of global LV filling curves. Abnormalities observed in the global LV filling curve may be less specific to the pathomechanism of diastolic dysfunction in the individuals with HFpEF when compared to GLS and Tau.

### Study limitations

Several limitations should be acknowledged in our study. First, the number of participants was relatively small. Small patient populations due to restrictive inclusion and exclusion criteria can lead to a narrow spectrum of myocardial conditions. Second, LA strain was only assessed from the 4-chamber view. Generally, 4-chamber view is susceptible to the accuracy of the breath-hold. However, the status of the breath-hold was highly stable in all HFpEF patients. The image quality of LA in the 2-chamber view cine CMR images was suboptimal in 7 patients with HFpEF (38.9%) in our study. Therefore, we used only the 4-chamber cine CMR to obtain LA strain.

## Conclusions

CMR-FT is a noninvasive approach that enables identification of the subgroup of HFpEF patients who have impaired LV GLS, and LV GLS assessed by this technique independently predicts abnormal LV relaxation measured by invasive conductance catheter in HFpEF patients. Diastolic dysfunction can be evaluated noninvasively in patients with HFpEF via the CMR approach using LV GLS in conjunction with ECV, which was previously shown to be an imaging biomarker for predicting LV stiffness.

## Data Availability

Not applicable.

## Abbreviations

BNP: Brain natriuretic peptide
BSA: Body surface area
CAD: Coronary artery disease
CMR: Cardiovascular magnetic resonance
CMR-FT: Cardiovascular magnetic resonance feature tracking
DICOM: Digital Imaging and Communications in Medicine
Ea: Arterial elastance
ECV: Extracellular volume fraction
EDP: End-diastolic pressure
EDVI: End-diastolic volume index
EF: Ejection fraction
eGFR: Estimated glomerular filtration rate
ESP: End-systolic pressure
ESPVR: End-systolic pressure–volume relationship
ESVI: End-systolic volume index
FA: Flip angle
FOV: Field of view
FT: Feature tracking
GCS: Global circumferential strain
Gd-DOTA: Gadoterate meglumine
GLS: Global longitudinal strain
GRS: Global radial strain
HFpEF: Heart failure with preserved ejection fraction
HR: Heart rate
LA: Left atrium/left atrial
LGE: Late gadoliniumenhancement
LGE: Late gadolinium-enhancement
LV: Left ventricular
MOLLI: Modified Look-Locker inversion recovery
nPFR: PFR normalized by LV end-diastolic volume
PCWP: Pulmonary wedge pressure
PFR: Peak filling rate
RA: Right atrium/right atrial
RV: Right ventricle/right ventricular
SD: Standard deviation
SENSE: Sensitivity encoding
TE: Echo time
TR: Repetition time
